# Decompressive laparotomy with temporary abdominal closure versus percutaneous puncture with placement of abdominal catheter in patients with abdominal compartment syndrome during acute pancreatitis: background and design of multicenter, randomised, controlled study

**DOI:** 10.1186/1471-2482-10-22

**Published:** 2010-07-12

**Authors:** Dejan V Radenkovic, Djordje Bajec, Nenad Ivancevic, Vesna Bumbasirevic, Natasa Milic, Vasilije Jeremic, Pavle Gregoric, Aleksanadar Karamarkovic, Borivoje Karadzic, Darko Mirkovic, Dragoljub Bilanovic, Radoslav Scepanovic, Vladimir Cijan

**Affiliations:** 1 Center for Emergency Surgery, Clinical Center of Serbia and School of Medicine, University of Belgrade, Belgrade, Serbia; 2 Institute for Anaesthesiology, Clinical Center of Serbia and School of Medicine, University of Belgrade, Belgrade, Serbia; 3 Institute for Medical Statistics, School of Medicine, University of Belgrade, Belgrade, Serbia; 4 Surgical Department, Military-Medical Academy, Belgrade, Serbia; 5 Surgical Department, Clinical Center "Bezanijska Kosa" and School of Medicine, University of Belgrade, Belgrade, Serbia; 6 Surgical Department, Clinical Center "Dr Dragisa Misovic," and School of Medicine, University of Belgrade, Belgrade, Serbia; 7 Surgical Department, Clinical Center "Zvezdara" and School of Medicine, University of Belgrade, Belgrade, Serbia

## Abstract

**Background:**

Development of abdominal compartment syndrome (ACS) in patients with severe acute pancreatitis (SAP) has a strong impact on the course of disease. Number of patients with this complication increases during the years due more aggressive fluid resuscitation, much bigger proportion of patients who is treated conservatively or by minimal invasive approach, and efforts to delay open surgery. There have not been standard recommendations for a surgical or some other interventional treatment of patients who develop ACS during the SAP. The aim of DECOMPRESS study was to compare decompresive laparotomy with temporary abdominal closure and percutaneus puncture with placement of abdominal catheter in these patients.

**Methods:**

One hundred patients with ACS will be randomly allocated to two groups: I) decompresive laparotomy with temporary abdominal closure or II) percutaneus puncture with placement of abdominal catheter. Patients will be recruited from five hospitals in Belgrade during two years period. The primary endpoint is the mortality rate within hospitalization. Secondary endpoints are time interval between intervention and resolving of organ failure and multi organ dysfunction syndrome, incidence of infectious complications and duration of hospital and ICU stay. A total sample size of 100 patients was calculated to demonstrate that decompresive laparotomy with temporary abdominal closure can reduce mortality rate from 60% to 40% with 80% power at 5% alfa.

**Conclusion:**

DECOMPRESS study is designed to reveal a reduction in mortality and major morbidity by using decompresive laparotomy with temporary abdominal closure in comparison with percutaneus puncture with placement of abdominal catheter in patients with ACS during SAP.

**Trial registration:**

ClinicalTrials.gov Identifier: NTC00793715

## Background

There is growing evidence in the literature that development of abdominal compartment syndrome (ACS) in patients with severe acute pancreatitis (SAP) has a strong impact on the course of disease [1-4]. The main causes for development of ACS during the course of SAP are: pancreatic and peripancreatic inflammation, visceral oedema caused by aggressive fluid resuscitation, presence of free fluid collection, and paresis of the bowel. Several studies clearly showed that development of organ failure in SAP is in correlation with presence of intraabdominal hypertension (IAH) [2,3,5,6]. It seems that the number of patients with this complication has increased, due to more aggressive fluid resuscitation, a much bigger proportion of patients treated conservatively or by a minimal invasive approach, and efforts to delay open surgery.

Intra-abdominal hypertension reduces organ perfusion and may cause organ dysfunction [7,8]. Increased intra-abdominal pressure (IAP) leads to hypoperfusion of the gastrointestinal tract and reduction of chest wall compliance [9]. It has also been shown that an IAP above 20 mmHg can lead to oliguria and significant reduction in the cardiac output [10,11]. IAH was associated with significantly higher APACHE II score and MODS score in patients with SAP [3,4]. De Walle et al. [5] published a higher incidence of respiratory, circulatory and renal failure among the patients with IAH. In patients with severe acute pancreatitis, pancreatic perfusion is reduced, and IAH probably contributes to further development of pancreatic hypoperfusion and consequently necrosis.

Some recent studies suggested that ACS is a frequent finding in patients with SAP [3,6,8,12,13]. Tao et al. [8] reported a 36% incidence of ACS among 297 patients with SAP. In a recently published study Al-Barhani et al. [3] showed an incidence of 61% of IAH and 56% of ACS in a selected well-studied and monitored group of SAP patients. However, the lack of a definition of ACS and methodological issues, make interpretation of these results and some other studies difficult.

So far, there have not been standard recommendations for a surgical or some other interventional treatment of patients who develop ACS during the course of SAP [14]. Despite the fact that World Society of Abdominal Compartment Syndrome (WSACS) published definition of IAH and ACS [15] and recommendation for the treatment [16], the appropriate surgical technique for the treatment of those patients suffering from SAP is still debated. Some procedures have been occasionally reported that could be useful and may be able to improve outcome of patients who develop ACS during SAP. Several authors published relief of ACS after insertion of drain under radiological guidance [12,17-19]. Some others recommended decompressive laparotomy with subsequent laparostomy for the treatment of ACS [1,8,20-22]. Several investigators also suggested skin incisions to perform a subcutaneous fasciotomy with the peritoneum left intact [23,24].

Sun et al. [12] performed a randomised study to compare effects of indwelling catheter and conservative measures in the treatment of ACS in fulminant acute pancreatitis. They found that drainage volume was positively correlated with intraabdoninal pressure, which also was correlated with hospitalization time and APACHE II score. Effects of the treatment in the group with abdominal catheter were significantly better than in conservative group, regarding relief of abdominal pain and hospitalization time. In addition mortality rate decreased from 20.7% to 10%, but without significant difference.

Decompressive laparotomy for ACS associated with SAP has not been studied in large patients group [14]. Occasionally, there have been several case reports in the literature with high early mortality rate, ranging from 17 to 75% [1-6,20-22,25]. A high proportion of patients in these reports, during surgical decompression received retroperitoneal debridement and early mortality was mainly associated with uncontrolled retroperiotoneal bleeding [5]. Current very limited experience supports the strategy of decompresive laparotomy in patients with ACS during SAP, but without premature exploration of pancreatic region and retroperitoneum [14].

All these data have not provided enough clear evidence to support a treatment algorithm for ACS in patients with SAP, although two approaches deserve more attention than other. These are decompresive laparotomy with temporary abdominal closure and percutaneous puncture with placement of abdominal catheter. Both of these procedures raise several unresolved issues such as for decompresive laparotomy: a) the relation to potential necrosectomy, b) difficulties in management of semi-open abdomen, c) increased risk of enteric fistulas d) potentially higher number patients with infected pancreatic necrosis than expected e) incidence of postoperative hernias. The main unanswered question for percutaneous puncture with placement of abdominal catheter is whether using this procedure is possible to achieve sufficient decompression and relief of ACS.

We anticipated that decompresive laparotomy with temporary abdominal closure, beside all potentially negative side effects that early open surgery carries in patients with acute pancreatitis, may result in decrease of overall mortality and major morbidity. The DECOMPRESS study is designed to compare effects of decompresive laparotomy with temporary abdominal closure and percutaneous puncture with placement of abdominal catheter in patients with abdominal compartment syndrome during acute pancreatitis.

## Methods

### Study objectives

The objective of this study is to compare outcome of two different interventions in the treatment of abdominal compartment syndrome in patients suffering from acute pancreatitis.

### Primary endpoint

The primary endpoint is mortality rate during the hospital stay for patients with acute pancreatitis.

### Secondary endpoints

Secondary endpoints are duration time of organ failure, development of "new organ failure", number of infectious complications, necrosectomy, enteric fistulas, intensive care stay, and total hospital stay.

### Participants

Patients will be enrolled from five university hospitals from Belgrade, Serbia.

### Inclusion criteria

The study population consists of patients with AP complicated by development of abdominal compartment syndrome. Organ dysfunction (Table 1) in association with intra-abdominal hypertension will define the presence of abdominal compartment syndrome. Intra-abdominal hypertension will be defined as an IAP of 20 mm Hg and higher.

**Table 1:** Definitions of organ failure

Organ Failure	Definition
Circulatory	Systolic blood pressure less than 90 mmHg or need for catecholamine support

Pulmonary	PaO2 60 mmHg or less or need for mechanical ventilation

Renal	Need for hemodialysis or creatinine level greater than 177 umol/L after rehydration

### Exclusion criteria

Patients will not be enrolled to the study if any of the following criteria are present: a) age < 18 and > 80 years; b) recent surgical interventions c) psychoses d) pregnancy e) previously history of chronic pancreatitis.

### Treatment and monitoring before the randomization

All patients with AP will be treated according to pre-established treatment protocol. The treatment protocol for patients with predicted SAP comprising: enteral nutrition preferably via a nasogastric route (in case of intolerance nasojejunal tube will be inserted), an early endoscopic retrograde cholagiopancreatography (ERCP) with sphincterotomy in patients with obstructive cholangitis and/or jaundice and antibiotic prophylaxis. If gastrointestinal nutrition is contraindicated, the patients will receive parenteral nutrition. Patients with predicted mild form of disease will be treated with intravenous fluid and analgetics with close monitoring of vital signs, renal and pulmonary function. Intraabdominal pressure will be monitored routinely on daily basis in all patients with predicted SAP, represented by at least one of the following criteria: APACHE II score ≥ 8, Imrie score ≥ 3 points and CRP ≥ 150 mg/L. In addition, in all patients with clinically suspected intraabdominal hypertension, abdominal pressure will be also measured daily. In all patients who have diagnosed IAH or predicted SAP, SOFA score will be calculated daily. It is well known that, usually abdominal compartment syndrome develops in the very early course of disease (during the first week) and the we expect that vast majority of our patients will be recruited within that time frame. Although, if there will be patients with ACS in later course it will not be an exclusion criteria. Since, many patients will be transferred from referring centres, violation of pre-randomization protocol is not an exclusion criteria.

Intravesical pressure, which indirectly reflects IAP, will be measured using a standard technique that has been described by Cheatham et al. [26] Contrast enhanced CT (CCT) will be done in all patients with IAH, immediately after the diagnosis has been established. This diagnostic toll will be also performed after 7-10 days an all patients with predicted SAP.

Patients with abdominal compartment syndrome will be randomised either for decompressive laparotomy with temporary abdominal closure or percutaneous puncture with placement of abdominal catheter.

### Group A: Decompresive laparotomy with temporary abdominal closure

Laparotomy will be performed via midline incision extending from xyphoid processus until pubis. If ascites is present, it will be evacuated and sent for biochemical and microbiological analysis. Abdominal cavity will be carefully inspected, but any manipulation on necrotic area and lesser sac are not allowed. After cleaning the abdomen, one large bore drain will be placed in the Douglas space. Abdomen will be temporarily closed using an abdominal zipper placed on fascial edges. Abdominal pressure will be measured one hour after surgery and every day at 9 a.m. during next 10 days. Planned staged reoperation will be repeated at 72 h intervals and if it is technically possible abdomen will be definitively closed after 3 reoperation or 9 days, independently of clinical picture and patients conditions. If there is clinical improvement and IAP decreases bellow 15 mm Hg, the abdomen wall will be definitively reconstructed and drain will be taken out, before that period of time. "Clinical improvement" is defined as: no more need for mechanical ventilation, dialysis/hemofiltration and catecholamines for at least 2 days.

### Group B: Percutaneous puncture with placement of abdominal catheter

Insertion of abdominal catheter diameter will be performed percutaneously 2 cm bellow the umbilicus. Fluid collected during drainage will be sent for biochemical and microbiological analysis. The abdominal catheter will be removed after 9 days. Measurement of intraabdominal pressure will be done in the same time interval as in group A.

### Management after the intervention

Post interventional management will be similar in both groups. All necessary intensive care measures will be continued and SOFA score will be calculated daily. In patients with further clinical deterioration or with clinical signs of infection/sepsis careful microbiological and CCT examination will be performed. After exclusion of other infection causes such as urinary tract, lungs and intravenous catheters, ultrasound guided fine-needle aspiration (FNA) of the pancreatic necrosis with consecutive Gram stain and bacteriologic culture will be carried out. If the infection of pancreatic necrosis is suspected, either by positive microbiological FNA findings or presence of gas in necrotic area on CCT, surgical intervention will be indicated. If the presence of infected necrosis is suspected during first 2 weeks of treatment, surgery may be postponed until third week of treatment. Standard surgical procedure will be maximal necrosectomy followed by continuous closed lavage. During surgery, at least two smears for bacteriologic culture will be taken from the area of pancreatic/peripancreatic necrosis.

### Data collection and follow-up

This study is coordinated by Emergency Surgical Department, Clinical Center of Serbia and School of Medicine, University of Belgrade. The clinical data of all randomized patients will be collected centrally in a database at the Emergency Surgical Department, Clinical Center of Serbia and School of Medicine, University of Belgrade. Trial coordinator will be in regular contact with the participating centers and will monitor the data of the every randomized patient.

Patients will be monitored during their hospital stay. For both, primary and secondary endpoints, follow-up will be finished at hospital discharge or death. Routine follow up visit are planned 3 and 6 month after discharge, including physical examination, abdominal ultrasound and/or CCT and/or MRI examination, to exclude presence of incisional hernias and pancreatic pseudocyst or other post-pancreatitis complications.

### Randomization

**Figure 1:**
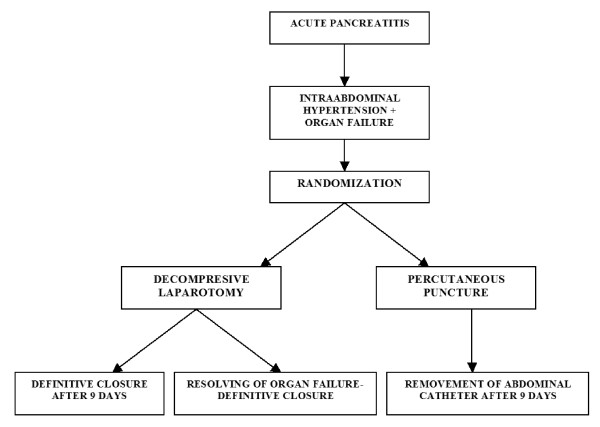
Flowchart with plan of randomization

The study statistician generated and administrated 1:1 allocation randomization arrangement for the 5 study hospitals. Patients will be randomly assigned to group A (decompressive laparotomy with temporary abdominal closure) or group B (percutaneous puncture with placement of abdominal catheter) as shown in the flow-chart (Figure 1.). We did not stratify hospitals by procedure-volume or teaching status since all hospitals are under supervision of Medical Faculty of University of Belgrade, and the assumption is that hospitals are similar.

### Statistical analysis

The analysis will be performed on the basis of an intention to treat (ITT) population and with respect to ITT principles.

The primary analysis consists of a comparison of the mortality rate during the hospital stay for acute pancreatitis in both treatment strategies. The comparison will be expressed in terms of relative risk and 95% confidence intervals. The research hypothesis will be evaluated by Fisher's exact test or Hi-square test (where appropriate), with a two-sided 0.05 significance level. In addition, survival probabilities over time will be estimated by Kaplan-Meier method and groups will be compared using the long-rank test.

Secondary endpoints as duration time of organ failure, intensive care and total hospital stay will be evaluated by t-test, while complication rates will be evaluated by Fisher's exact test or Hi-square test (where appropriate), also with a two-sided 0.05 significance level.

### Sample size calculation

The sample size calculation is based on equivalence design for the primary endpoint- mortality rate of patients with abdominal compartment syndrome during acute pancreatitis. It is anticipated that decompressive laparotomy with temporary abdominal closure will lead to a mortality rate reduction from 60% of patients to 40%. Therefore, the expected reduction in mortality that was used for sample size calculation is 20%. With calculated 80% power of the study (beta error 0.20) and alpha error of 0.05 (two-sided) complete data from 47 patients per group will be necessary to demonstrate this effect, if it truly exists. Taking into account a 7% loss-to-follow up, a total of 2 × 50 patients have to be randomized.

Reported annual rate of treated patients with severe form of acute pancreatitis is about 250 (based on hospital data of Emergency Centre, Clinical Centre of Serbia, 3 affiliated hospitals of School of Medicine University of Belgrade and Military Academy), while the prevalence of abdominal compartment syndrome in this patient population is about 20%. Consequently, the total expected number of patients with abdominal compartment syndrome during acute pancreatitis in these 5 centres is about 50, annually. Based on this assumption, the expected study inclusion period would be ended in two years.

## Discussion

The primary objective of present study is to compare two approaches aiming to try to determine the role of ACS in patients with SAP. During the last 15 years early surgical intervention in patients with SAP was not recommended and conservative treatment is a gold standard. Since recently, ACS during SAP attracted more attention and those patients are candidates for some type of intervention [14]. Surgical decompression may be the optimal solution [1,3,20-22,25], but mortality still remains high and it is resource-consuming and labor-intensive procedure, accompanied with increased risk of enteric fistulas, hemorrhaging, and possible greater incidence of infection of necrosis [5]. Percutaneous puncture with placement of abdominal catheter eliminates surgical intervention and open abdomen, but might not be sufficient in these very sick patients [3,4].

The very important step in the treatment of the ACS is the early prevention of the harmful effects [2-4,8,12-14]. Some of type of decompression should be performed as soon as diagnosis of ACS is established [14,16]. Although in routine clinical practice controversies persist regarding the timing, type of decompression procedure, and indications in patients with SAP [14]. Several investigators suggested that prompt recognition of ACS and adequate treatment could be crucial [3,4,14,16].

There are suggestions in the literature that percutaneous drainage should be the first step in the treatment of the patients with IAH and ACS [3,4,6,12,14,17-19]. Only patients who do not respond to this type of treatment should be candidate for surgical decompression [3,4,6,8,12]. Bearing in mind the high mortality rate among those patients, it remains unclear whether earlier surgical decompression will be able to reduce it. We hypothesized that open surgery, immediately after the diagnosis of ACS is established, could improve outcome of this patients. It is very important to stress that according to the study protocol, during the surgical intervention the retroperitoneum will be left untouched in order to avoid uncontrollable bleeding and possible opening of the route for infection of the necrotic area.

## Conclusions

Currently, there are no prospective randomized trials in patients with ACS during SAP. There are several reasons including: low incidence, low hospital volumes with patients with AP, individualised treatment approach and very heterogeneous type of disease. Therefore we have planned this multicenter study in one city which serves a region of 3 million inhabitants. With a very similar treatment algorithm, these centres easily adapted their practice to the protocol for this study.

The final version of study protocol was approved by Ethical Committee Clinical Center of Serbia on 9^th ^of June, 2010.

## Competing interests

The authors declare that they have no competing interests.

## Authors' contributions

DVR drafted the manuscript.

DJB and NI co-authored the writing of the manuscript.

DVR, DJB, NI, VB, VJ, PG, BK, AK, DM, DB and VC participated in the design of the study during several meetings of the study group.

NM performed all statistical calculations.

All authors edited the manuscript and read and approved the final manuscript.

## Pre-publication history

The pre-publication history for this paper can be accessed here:

http://www.biomedcentral.com/1471-2482/10/22/prepub

## References

[B1] Gecelter G Fahoum B Gardezi S Schein M Abdominal compartment syndrome in severe acute pancreatitis: an indication for a decompressing laparotomy? Dig Surg 2002 19 402 404 10.1159/000065820 12435913

[B2] Chen H Li F Sun JB Jia JG Abdominal compartment syndrome in patients with severe acute pancreatitis in early stage World J Gastroenterol 2008 14 3541 3548 10.3748/wjg.14.3541 18567084 PMC2716618

[B3] Al-Bahrani AZ Abid GH Holt A McCloy RF Benson J Eddleston J Clinical relevance of intra-abdominal hypertension in patients with severe acute pancreatitis Pancreas 2008 36 39 43 10.1097/mpa.0b013e318149f5bf 18192879

[B4] Dambrauskas Z Parseliunas A Gulbinas A Pundzius J Barauskas G Early recognition of abdominal compartment syndrome in patients with acute pancreatitis World J Gastroenterol 2009 15 717 721 10.3748/wjg.15.717 19222096 PMC2653440

[B5] De Waele JJ Hoste E Blot SI Decruyenaere J Colardyn F Intra-abdominal hypertension in patients with severe acute pancreatitis Crit Care 2005 9 R452 R457 10.1186/cc3754 16137360 PMC1269467

[B6] Keskinen P Leppaniemi A Pettila V Piilonen A Kemppainen E Hynninen M Intra-abdominal pressure in severe acute pancreatitis World J Emerg Surg 2007 2 2 10.1186/1749-7922-2-2 17227591 PMC1800837

[B7] Malbrain ML Chiumello D Pelosi P Bihari D Innes R Ranieri VM Incidence and prognosis of intraabdominal hypertension in a mixed population of critically ill patients: a multiple-center epidemiological study Crit Care Med 2005 33 315 322 10.1097/01.CCM.0000153408.09806.1B 15699833

[B8] Tao HQ Zhang JX Zou SC Clinical characteristics and management of patients with early acute severe pancreatitis: experience from a medical center in China World J Gastroenterol 2004 10 919 921 15040047 10.3748/wjg.v10.i6.919 PMC4727019

[B9] Schwarte LA Scheeren TW Lorenz C De BF Fournell A Moderate increase in intraabdominal pressure attenuates gastric mucosal oxygen saturation in patients undergoing laparoscopy Anesthesiology 2004 100 1081 1087 10.1097/00000542-200405000-00009 15114204

[B10] Diebel LN Dulchavsky SA Brown WJ Splanchnic ischemia and bacterial translocation in the abdominal compartment syndrome J Trauma 1997 43 852 855 10.1097/00005373-199711000-00019 9390500

[B11] Pelosi P Brazzi L Gattinoni L Vincent JL Measuring intra-abdominal pressure in intensive care setting Yearbook of Intensive Care and Emergency Medicine 2001 Berlin, Germany: Springer-Verlag 586 595

[B12] Sun ZX Huang HR Zhou H Indwelling catheter and conservative measures in the treatment of abdominal compartment syndrome in fulminant acute pancreatitis World J Gastroenterol 2006 12 5068 5070 16937509 10.3748/wjg.v12.i31.5068 PMC4087416

[B13] Pupelis G Austrums E Snippe K Berzins M Clinical significance of increased intraabdominal pressure in severe acute pancreatitis Acta Chir Belg 2002 102 71 74 12051093 10.1080/00015458.2002.11679269

[B14] De Waele JJ Leppaniemi AK Intra-abdominal hypertension in acute pancreatitis World J Surg 2009 33 1128 1133 10.1007/s00268-009-9994-5 19350318

[B15] Malbrain ML Cheatham ML Kirkpatrick A Sugrue M Parr M De WJ Results from the International Conference of Experts on Intra-abdominal Hypertension and Abdominal Compartment Syndrome. I. Definitions Intensive Care Med 2006 32 1722 1732 10.1007/s00134-006-0349-5 16967294

[B16] Cheatham ML Malbrain ML Kirkpatrick A Sugrue M Parr M De WJ Results from the International Conference of Experts on Intra-abdominal Hypertension and Abdominal Compartment Syndrome. II. Recommendations Intensive Care Med 2007 33 951 962 10.1007/s00134-007-0592-4 17377769

[B17] Reed SF Britt RC Collins J Weireter L Cole F Britt LD Aggressive surveillance and early catheter-directed therapy in the management of intra-abdominal hypertension J Trauma 2006 61 1359 1363 10.1097/01.ta.0000245975.68317.5a 17159677

[B18] Reckard JM Chung MH Varma MK Zagorski SM Management of intraabdominal hypertension by percutaneous catheter drainage J Vasc Interv Radiol 2005 16 1019 1021 16002511 10.1097/01.RVI.0000157781.67279.72

[B19] Latenser BA Kowal-Vern A Kimball D Chakrin A Dujovny N A pilot study comparing percutaneous decompression with decompressive laparotomy for acute abdominal compartment syndrome in thermal injury J Burn Care Rehabil 2002 23 190 195 10.1097/00004630-200205000-00008 12032369

[B20] Wong K Summerhays CF Abdominal compartment syndrome: a new indication for operative intervention in severe acute pancreatitis Int J Clin Pract 2005 59 1479 1481 10.1111/j.1368-5031.2005.00658.x 16351683

[B21] Siebig S Iesalnieks I Bruennler T Dierkes C Langgartner J Schoelmerich J Recovery from respiratory failure after decompression laparotomy for severe acute pancreatitis World J Gastroenterol 2008 14 5467 5470 10.3748/wjg.14.5467 18803361 PMC2744173

[B22] Leppaniemi A Mentula P Hienonen P Kemppainen E Transverse laparostomy is feasible and effective in the treatment of abdominal compartment syndrome in severe acute pancreatitis World J Emerg Surg 2008 3 6 10.1186/1749-7922-3-6 18234076 PMC2266717

[B23] Leppaniemi AK Hienonen PA Siren JE Kuitunen AH Lindstrom OK Kemppainen EA Treatment of abdominal compartment syndrome with subcutaneous anterior abdominal fasciotomy in severe acute pancreatitis World J Surg 2006 30 1922 1924 10.1007/s00268-006-0024-6 16983467

[B24] Cheatham ML Fowler J Pappas P Subcutaneous linea alba fasciotomy: a less morbid treatment for abdominal compartment syndrome Am Surg 2008 74 746 749 18705579

[B25] De Waele JJ Hesse UJ Life saving abdominal decompression in a patient with severe acute pancreatitis Acta Chir Belg 2005 105 96 98 15790212

[B26] Cheatham ML Safcsak K Intraabdominal pressure: a revised method for measurement J Am Coll Surg 1998 186 594 595 10.1016/S1072-7515(98)00122-7 9583702

